# Redefining *Primum Non Nocere* to Include Reproductive Autonomy: A New Paradigm in Subspecialty Medicine

**DOI:** 10.1089/whr.2021.0079

**Published:** 2021-10-26

**Authors:** Mehret Birru Talabi, Lisa S. Callegari, Sonya Borrero

**Affiliations:** ^1^ Division of Rheumatology and Clinical Immunology, Center for Women's Health Research and Innovation, Center for Research on Health Care, Department of Medicine, University of Pittsburgh, Pittsburgh, Pennsylvania, USA.; ^2^ Health Services Research and Development, Seattle-Denver Center of Innovation for Veteran-Centered and Value-Driven Care, Department of Veterans Affairs (VA) Puget Sound Health Care System, Seattle, Washington, USA.; ^3^ Department of Obstetrics and Gynecology, University of Washington School of Medicine, Seattle, Washington, USA.; ^4^ Department of Health Services, University of Washington School of Public Health, Seattle, Washington, USA.; ^5^ Division of General Internal Medicine, Center for Women's Health Research and Innovation, Department of Medicine, University of Pittsburgh School of Medicine, Pittsburgh, Pennsylvania, USA.; ^6^ Center for Health Equity, Research, and Promotion, VA Pittsburgh Health Care System, Pittsburgh, Pennsylvania, USA.

**Keywords:** chronic disease, maternal mortality, reproductive autonomy, shared decision-making, subspecialty medicine

## Abstract

People with chronic medical illnesses are at particularly high risk for adverse pregnancy outcomes, yet current clinical approaches largely fail to identify and support their individualized reproductive and pregnancy goals. Instead, the predominant approach to pregnancy in subspecialty medicine is disease centered rather than patient centered. To better meet the individual needs and preferences of people with childbearing potential who have chronic medical conditions, we advocate in this article for a paradigm shift in subspecialty care that honors individuals' reproductive autonomy and human right of reproduction.

## Introduction

Dismal rates of maternal morbidity and mortality in the United States have sparked widespread interest in critically examining and transforming existing reproductive health care practices and systems.^1^ Chronic medical illness has been implicated as a leading cause of poor maternal health outcomes.^2^ Subspecialty medicine clinicians have traditionally discouraged patients with serious chronic illnesses from pursuing pregnancy to prevent adverse pregnancy-associated health risks—a practice that may rise with the current spotlight on maternal outcomes. Deeper interrogation of this tendency on the part of subspecialty providers reveals an important and perhaps overlooked ethical and moral tension: the motivation to prevent adverse outcomes on a public health level may undermine people's reproductive autonomy at the individual level. In light of the current national reckoning on equity and social justice in medicine, we believe that a paradigm shift in subspecialty care is warranted to center individuals' pregnancy preferences and honor their fundamental human right to reproduce, through which their pregnancy and perinatal outcomes may be improved.

## Pregnancy-Associated Risks Among People with Chronic Medical Illnesses

Pregnancies among people with chronic and complex medical illnesses, such as systemic lupus erythematosus, pulmonary hypertension, diabetes, and chronic kidney disease, are more likely to be complicated by pre-eclampsia, intrauterine growth restriction, and maternal and/or fetal death than among healthy people.^2,3^ As reported across many different diseases, most patients whose illnesses are well controlled with safe medications for at least several months before pregnancy have better maternal and fetal outcomes than people whose diseases are poorly controlled at the time of pregnancy. This underscores the importance of family planning care as a way in which clinicians may optimize health and reproductive outcomes to meet patients' needs related to pregnancy or pregnancy prevention. However, the extent to which a clinician meets a patient's needs may vary, particularly when the clinician's advice is not aligned with the patient's goals or preferences.

## Challenges in Subspecialty Family Planning Care

One of the key principles of medical ethics is the dictum of *primum non nocere—*first, do no harm. Ironically, an impulse to protect pregnant people and fetuses in medical research has perhaps put them at even great risk by systemically excluding them from studies and thereby precluding an evidence base with which to support informed decision-making and sound clinical recommendations across a range of reproductive states or decisions (*e.g.*, pregnancy, contraception, fertility, and abortion). The potential for diseases to become more severe and life-threatening in the context of pregnancy, and lack of evidence-based guidance for treating disease in pregnancy, have contributed to a culture in subspecialty medicine that centers the disease and clinical outcomes above more holistic and humanistic approaches to family planning. This culture generally privileges pregnancy prevention over pregnancy, and contraception efficacy and safety above a patient's preferences for or even interest in using contraception.^4^

Subspecialty clinicians are tasked with determining a patient's disease-related pregnancy risks and providing appropriate family planning counseling. It is not surprising that narrative studies reveal that many subspecialty clinicians are anxious about even the potential of managing high-risk pregnancies.^5^ Clinicians may feel that to encourage such pregnancies might violate the principle of *primum non nocere.* Clinicians also shoulder the burden of managing patients' pregnancies and perinatal complications in the clinical setting, often with limited data, time, and resources. Concerns about their expertise in managing high-risk pregnancy, patients' welfare, and the professional, psychological, and legal consequences of poor outcomes may lead clinicians to encourage people with serious illnesses to avoid pregnancy altogether.^5^ Moreover, clinicians are not immune to social norms and biases regarding who is deemed “worthy” of childbearing, and impulses to constrain reproduction among people with chronic medical illnesses may be further amplified among those who are additionally socially or economically disadvantaged.^6^

The increased visibility of high rates of U.S. maternal morbidity and mortality may provide additional rationale for clinicians who are already inclined to discourage reproduction among people with chronic medical conditions. We acknowledge that the more people with chronic medical illnesses pursue pregnancy, the more maternal and neonatal deaths may result. However, a majority of these pregnancies, although potentially complex, can be carried and end safely and well with appropriate management and support. Moreover, for many people, family formation through childbearing is a central aspect of their humanity, identity, and dignity. Thus, some may wish to pursue pregnancy with full acceptance of the health risks, perhaps reflected in the steadily rising rates of pregnancy among people with chronic medical illnesses.^2^ People who sense that their clinicians would disagree with or judge their reproductive goals may be rightfully reluctant to disclose their true preferences or intentions around reproduction. In the absence of a supportive patient–clinician relationship, the opportunity to help a patient to prevent an undesired pregnancy or to mitigate a patient's key health risks before pregnancy (*e.g.*, transitioning from a teratogen to a safer medication or providing contraception to delay pregnancy until a period of disease quiescence) is lost.

## Shared Reproductive Decision-Making in Subspecialty Care

We propose that a different approach to pregnancy and family planning will better serve the needs of people with childbearing capacity who have chronic medical illnesses. For the past 20 years, scholars and advocates have promoted a model for family planning care guided by principles of human rights, reproductive justice, and autonomy.^7^ This approach recognizes the importance of procreative liberty as both an individual right and a matter of social justice. By fully embracing this approach in health care, the medical community can begin to reverse paternalistic instincts that have undermined the reproductive autonomy of many subgroups of people, including Black people and other people of color, as well as those who are low income, disabled, or whose physical or mental fitness to reproduce is deemed as suboptimal.^8^

We advocate for a model of care in subspecialty medicine that is person centered rather than disease centered, and is supported by shared decision-making principles. Clinicians must create an environment in which patients feel comfortable articulating their thoughts and feelings about pregnancy, even if incompletely formulated, and in which clinicians offer insights about patients' current health status, information about how the disease and medications might affect a pregnancy, and recommendations about pregnancy timing in the disease context. Clinicians will share relevant and personalized information with the patient but will not attempt to influence the patient's reproductive decisions. Rather, shared decision-making in this context is intended to support patients in making informed decisions about the full range of their reproductive options, and to achieve their *own* reproductive goals as safely as possible.

We also suggest that clinicians initiate family planning discussions with every patient with childbearing potential, using open-ended, nondirective, and nonjudgmental language that elicits their preferences for family formation and timing. At the very least, we propose that clinicians initiate these conversations at the first clinical encounter, whenever medications are initiated or changed, and when a person's disease is active or severe. Subspecialists should also build relationships with clinicians in primary care, maternal fetal medicine, and family planning to create accessible pathways for the timely management of patients with urgent needs for contraception, pregnancy care, or abortion. These patient-centered approaches may help to better anticipate patients' needs and mitigate health risks while simultaneously centering their reproductive autonomy.

## Conclusions

The current spotlight on the U.S. maternal morbidity and mortality crisis requires an evolving paradigm that addresses family planning care in the subspecialty context. Many people with chronic medical illnesses will have high-risk pregnancies. However, a disease diagnosis does not and should not compromise the fundamental human right of reproductive freedom. We believe that incorporating patient-centered approaches to family planning in subspecialty care offers an important initial step toward supporting people with chronic and complex medical illnesses to safely achieve the reproductive goals that are right for them and for their families.
